# Color Stability of New Esthetic Restorative Materials: A Spectrophotometric Analysis

**DOI:** 10.3390/jfb8030026

**Published:** 2017-07-06

**Authors:** Claudio Poggio, Lodovico Vialba, Anna Berardengo, Ricaldone Federico, Marco Colombo, Riccardo Beltrami, Andrea Scribante

**Affiliations:** Department of Clinical, Surgical, Diagnostic and Pediatric Sciences–Section of Dentistry, University of Pavia, Pavia 27100, Italy; lodovico.vialba01@universitadipavia.it (L.V.); annaberardengo@libero.it (A.B.), federico.ricaldone01@universitadipavia.it (R.F.); marco.colombo@unipv.it (M.C.); riccardo.beltrami01@universitadipavia.it (R.B.); andrea.scribante@unipv.it (A.S.)

**Keywords:** CIE Lab, color stability, esthetic restorative materials

## Abstract

The aim of this in vitro study was to evaluate and compare the color stability of different esthetic restorative materials (one microfilled composite, one nanofilled composite, one nanoceramic composite, one microfilled hybrid composite, one microfilled hybrid composite, one nanohybrid Ormocer based composite and one supra-nano spherical hybrid composite) after exposure to different staining solutions (physiological saline, red wine, coffee). All materials were prepared and polymerized into silicon rings (2 mm × 6 mm × 8 mm) to obtain specimens identical in size. Thirty cylindrical specimens of each material were prepared. Specimens were immersed in staining solutions (physiological saline, coffee and red wine) over a 28-day test period. A colorimetric evaluation according to the CIE L*a*b* system was performed by a blind trained operator at 7, 14, 21, 28 days of the staining process. The Shapiro–Wilk test and ANOVA were applied to assess significant differences among restorative materials. A paired *t*-test was applied to test which CIE L*a*b* parameters significantly changed after immersion in staining solutions. All restorative materials showed significant color differences after immersion in coffee. Coffee caused a significant color change in all types of tested composite resins. Only Filtek Supreme XTE demonstrated a staining susceptibility to red wine; no other significant differences among the materials were demonstrated. Long-term exposure to some food dyes (coffee in particular) can significantly affect the color stability of modern esthetic restorative materials regardless of materials’ different compositions.

## 1. Introduction

Composite resins play an essential role in dental restoration, mostly in the anterior sector, not only because of its esthetic proprieties but also for the good maneuverability and the acceptable biocompatibility [1,2]. A crucial property of esthetic restorative materials is their long-term color stability and an unacceptable color match is a primary reason for replacement of composite resin restoration [2]. Due to their good aesthetic properties, resin composite materials are widely used in clinical practice. Any aesthetic restorative material should duplicate the appearance of a natural tooth in color, and the success of an aesthetic restoration depends first on the color match and then on the color stability of the material [3]. However, a major disadvantage is the staining and discoloration of the restorative material after prolonged exposure to the oral environment. This is due to the aging process in the oral environment. 

Three types of discolorations are generally described. (I) External discoloration, which is due to the accumulation of plaque and surface stains (extrinsic stain). (II) Surface or sub-surface color alteration, which implies superficial degradation or slight penetration and reaction of staining agents within the superficial layer of composite resins (absorption). (III) Body or intrinsic discoloration, which is due to physicochemical reactions in the deeper portion of the restoration [4]. Discoloration of composite resin materials can be caused by intrinsic and/or extrinsic factors [5]. Extrinsic discoloration is mainly caused by colorants contained in beverages and foods or beverage, smoking and poor oral hygiene are often associated to insufficient polymerization [6,7]. Various in vitro studies have demonstrated that common drinks and food ingredients could cause significant change in the surface color of resin composites [6,8]. If the stains are superficial, polishing the surface of the material is enough to restore initial esthetic overlook, otherwise is necessary to replace the whole restoration [9].

The aim of this in vitro study was to evaluate and compare the color stability of different esthetic restorative materials (one microfilled composite, one nanofilled composite, one nanoceramic composite, one microfilled hybrid composite, one microfilled hybrid composite, one nanohybrid Ormocer based composite and one supra-nano spherical hybrid composite) after exposure to different staining solutions (coffee, red wine). The null hypothesis is that esthetic restorative materials do not change color clinically when staining agents are routinely applied. 

## 2. Results

**Figure 1:**
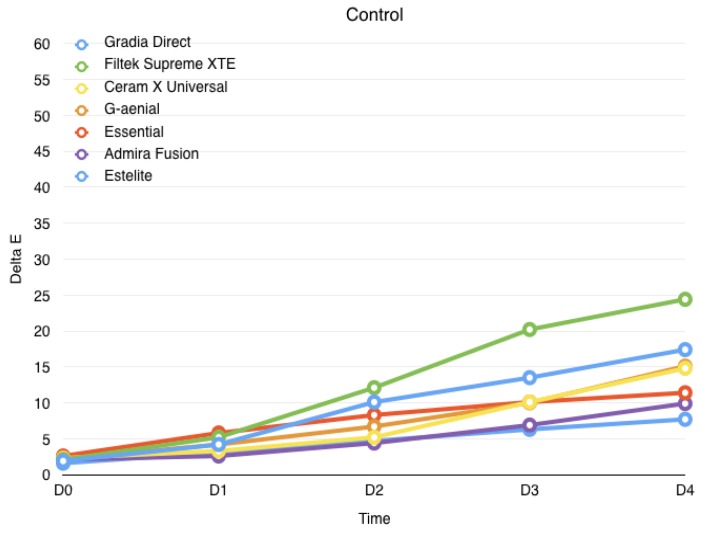
Evolution of the color variation for each material over the course of the study when immersed in control solution. 1 day (D0), 1 week (D1), 2 weeks (D2), 3 weeks (D3), 4 weeks (D4).

**Figure 2:**
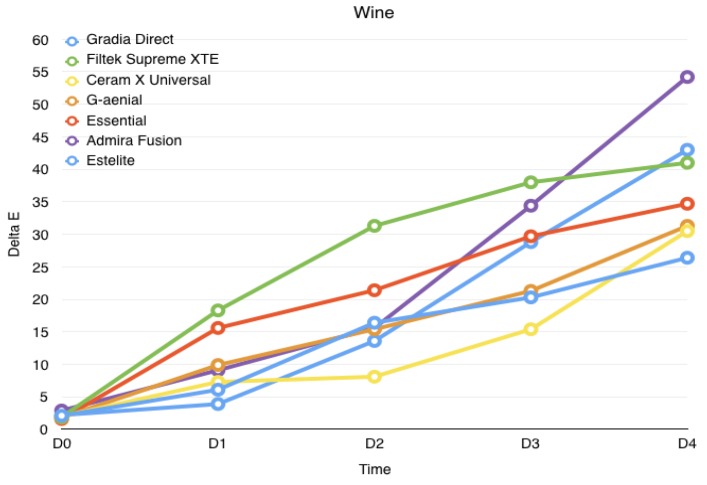
Evolution of the color variation for each material over the course of the study when immersed in wine. 1 day (D0), 1 week (D1), 2 weeks (D2), 3 weeks (D3), 4 weeks (D4).

**Figure 3:**
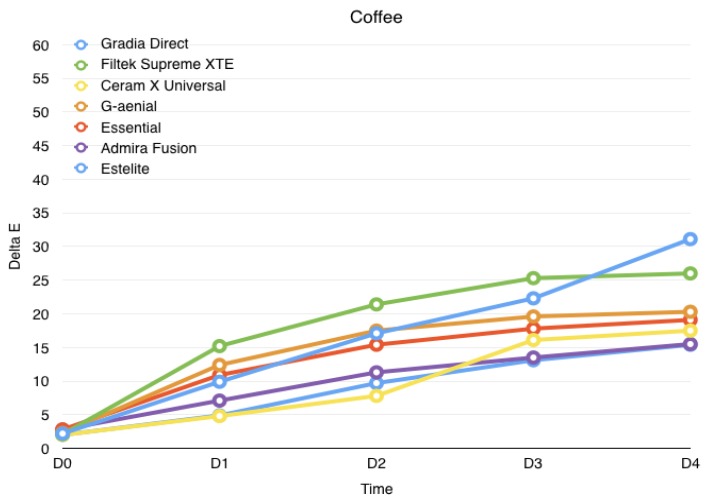
Evolution of the color variation for each material over the course of the study when immersed in coffee. 1 day (D0), 1 week (D1), 2 weeks (D2), 3 weeks (D3), 4 weeks (D4).

Results are summarized in Table 1 and displayed in Figure 1, Figure 2 and Figure 3. The Shapiro–Wilk test showed that the values were normally distributed. ANOVA found significant differences among the various groups. At the end of the study (4 weeks immersion), the highest discolorations were reported with wine (*p* > 0.05), significantly lower discolorations were reported with coffee (*p* < 0.05), whereas the lowest discolorations were reported for control groups (*p* < 0.05) for all materials tested. 

Immersion in coffee mainly affected the color stability of Gradia Direct Anterior, Filtek Supreme XTE, G-aenial and Essentia Enamel (*p* < 0.05). Immersion in physiological solution did not provide significant variations in CIE L*a*b* parameters for all the composites tested. After one month immersion, Filtek Supreme XTE and Admira Fusion showed the highest level of discoloration when immersed in physiological solution (*p* < 0.05). No perceivable discoloration was recorded for Gradia Direct Anterior when immersed in wine (ΔE < 3.3), while the alteration to other composite materials was comparable (*p* > 0.05). Immersion in coffee showed low ΔE values for Filtek Supreme XTE and G-aenial (ΔE < 3.3), while the other composite materials showed a similar discoloration rate ΔE > 3.3 (*p* > 0.05).

In the control group (physiologic solution), when comparing discoloration over time, the color variation is significant after three weeks for Gradia Direct and Filtek Supreme XTE (*p* < 0.05), whereas other groups showed a significant increase only after 4 weeks (*p* < 0.05).

After wine storage, the color variation is significant for Filtek Supreme XTE and Essentia composites after 1-week storage (*p* < 0.05). Other materials showed a significant increase after 2-week storage (*p* < 0.05).

After coffee storage, discoloration is significant after one week for Filtek Supreme XTE, G-Aenial, Essential and Gradia Direct. Other materials showed a significant increase after 2-week storage (*p* < 0.05).

## 3. Discussion

The null-hypothesis of the present investigation has been rejected. In the present study, significant differences were reported among various composites tested and among different staining solutions. This is in agreement with previous reports that tested other materials [10,11,12,13,14,15,16,17,18,19].

The importance of dental restoration color stability is crucial for dental professionals and patients. In fact, the quality of a restoration is considered from both functional and aesthetic points of view. The search for ideal esthetic and functional restoratives resulted in significant improvements in materials and techniques over time. Conventional dental composites showed average particle sizes of 1 µm, and typically have fillers close to 50 µm [2]. The introduction of fillers enhanced mechanical properties but increased polish difficulty [3]. Therefore, new nanofilled composites that contained only nanoscale particles were introduced [19].

In literature, many Authors evaluated the color stability of dental restorative materials using the CIE L*a*b* system [11,12,13,14,15,16,17,18]. The present investigation evaluated color stability over time (4 weeks) of seven different restorative materials. To our knowledge, in literature, there are no reports that evaluated and compared discoloration of micro and nano filled, nanoceramic, microfilled hybrid, Ormocer based and supra-nano hybrid composites. Previous reports tested other restorative materials showing different results. Ren et al. [11] studied the color stability of dental composite resins using the thermocycling stain challenge model, accounting for the complex effects of oral environment and tooth brushing. The composite resin discs were made with Filtek Supreme Ultra (FiltekSU), TPH3 and Renamel, and subjected to thermocycling challenges in warm coffee (55 °C/pH 5.2) and cold tea and fruit juice mixtures (5 °C/pH 3.6). They observed that color stability of FiltekSU is inferior to that of TPH3 and Renamel. The thermocycling stain challenge model can potentially differentiate surface staining that can be removed by brushing from true discoloration of the material that is refractory to oral hygiene procedures. In the present investigation, no thermocycling was applied, therefore, a direct comparison of the results is not possible.

Other authors [12] assessed the influence of commonly used types of coffee (American, Arabic, Turkish and Espresso coffee), on the surface microhardness and color stability of microhybrid resin-based composite (Filtek Z250), nanofilled resin-based composite (Filtek Supreme) and organic modified ceramic composite (Ormocer). All resin-based materials showed significant color change when compared to the control (saline). Filtek Z250 showed the least color change among the three materials followed by Ormocer. On the other hand, Filtek Supreme was the most common material prone to discoloration. Espresso coffee caused the most change in color followed by Turkish then American coffee. Nevertheless, Arabic coffee caused the least color change of the three materials. Additionally, in the present report, coffee showed significant discoloration of all the materials tested. 

Poggio et al. [13] evaluated the color stability of different restorative materials (one microfilled composite, one nanofilled composite, one nanohybrid composite and one Ormocer-based composite) after exposure to different staining solutions (coffee, coke and red wine). Coca cola and red wine did not influence the color stability for all restorative materials except for Filtek Supreme XTE. Coffee caused a significant color change in all types of tested composite resins. Filtek Supreme XTE demonstrated alone a staining susceptibility to red wine; no other significant differences among the materials were demonstrated. This is not in agreement with our report as wine caused the highest discoloration for all the materials tested, whereas coffee showed lower discoloration values. This is probably due to the different staining solution selection and to different material compositions.

However, long-term exposure to some food dyes (coffee and wine in particular) can significantly affect the color stability of modern esthetic restorative materials regardless of materials’ different compositions [13]. Other authors evaluated Microhybrid and nanohybrid composite resins and found similar in vitro surface discoloration in tea. After bleaching, discoloration was removed from some composite resins tested [14]. In the present report, the effect of bleaching has not been evaluated.

De Alencar et al. [16] evaluated the color and surface roughness of nanoparticle and nanohybrid composites after immersion in distilled water, acai juice, grape juice and red wine and repolishing. The specimens were reassessed after immersion for 1, 2, 4, 8, and 12 weeks and after repolishing. The results showed that after 2 weeks, there were statistically significant changes in color of both resins in all groups, with the exception of the specimens stored in distilled water. Red wine produced the greatest color change in nanocomposites, followed by grape juice. Acai juice made the color unacceptable clinically only after 12 weeks. Repolishing reduced the color change in all groups. In the present report, no repolishing was performed, however results obtained with wine were similar.

Llena et al. [17] evaluated the color stability of two nanohybrid resin-based composites, two organic modified ceramic resin composites (ormocers) and a compomer (GrandioSO, Esthet X, Dyract EXTRA, Ceram X duo, Admira Fusion) following their immersion for 4 weeks in four usual drinks: red wine, coffee, cola and distilled water. All the staining solutions produced darkening beyond clinically acceptable limits. Esthet X and Ceram X duo were the materials that experienced less staining, followed by GrandioSO. Admira Fusion and Dyract EXTRA were the more stained materials. Red wine was the drink that produced more staining, followed by coffee and cola. This is in agreement with the results of our report.

Erdemir et al. [18] studied the effects of three sports drinks on the color stability of two nanofilled and two microhybrid composite materials (Clearfil Majesty Posterior, Filtek Supreme, Clearfil APX, and Filtek Z250) after 1-month and 6-month periods. All the test solutions used in the study caused greater discoloration than the clinically acceptable level of the threshold over the 6-month evaluation period except for Clearfil Majesty Posterior immersed in distilled water. The effect of each solution on the color stability of the composite materials depended on the type of solution, exposure time, and composition of the composite material. 

In literature, other staining agents have also been studied. Energy drinks (Red Bull, Bison and Power Horse) have previously been tested on the color stability of nanofilled composite resins (Filtek Z350 XT, Tetric EvoCeram, and Filtek Z250 XT) after different periods of aging time (1, 7, 30, 60 days). The color change induced by Red Bull, Bison and Power Horse energy drinks was significantly different for all tested materials at all four times except in the Red Bull group. The highest total color difference was found in the Red Bull group after 60 days [19].

Our study observed that staining solutions (coffee and red wine) caused discoloration in every material tested. All the solutions, even distilled water, produced darkening beyond clinically acceptable limits, as shown in a previous study [17]. Admira Fusion showed the major discoloration caused by wine, followed by Filtek Supreme XTE. Gradia Direct Anterior and Filtek Supreme XTE were the materials more stained by coffee. G-aenial reported significant discoloration in all staining solutions. Therefore, color stability of resin-based materials is affected by their different material composition. On the basis of these considerations, further studies are needed in order to test future new materials available on the market.

## 4. Materials and Methods 

### 4.1. Specimens’ Preparation

One microfilled composite (Gradia Direct Anterior), one nanofilled composite (Filtek Supreme XTE), one nanoceramic composite (Ceram X Universal), one microfilled hybrid composite (G-aenial), one microfilled hybrid composite (Essentia Enamel), one nanohybrid Ormocer based composite (Admira Fusion) and one supra-nano spherical hybrid composite (Estelite) were evaluated in this study (Table 2). For each brand, the A2 Vita shade was selected. 

All materials were polymerized according to the manufacturers’ instructions into silicon rings (height 2 mm; internal diameter 6 mm; external diameter 8 mm) to obtain specimens identical in size. Cavities of these rings were slightly overfilled with material, covered with a transparent polyester film strip (Mylar strip, Henry Schein, Melville, NY, USA), pressed between glass plates and polymerized for 40 s on each side using a curing unit (Celalux II, Voco, Cuxhaven, Germany). One light polymerization mode was used for each material—standard: 1000 mW·cm^−2^ for 40 s. The intensity of the light was verified with a radiometer (SDS Kerr, Orange, CA, USA). The light was placed perpendicular to the specimen surface, at a distance of 1.5 mm. A total of forty specimens of each composite resin were prepared. 

### 4.2. Staining Process

The staining solutions used were: physiological solution (negative control), red wine (Bonarda Tenuta Casa Re, Montecalvo Versiggia/PV, Italy) and coffee (Nescafe Classic, Nestle, Vevey, Switzerland).

The specimens were immersed in staining solutions at room temperature over a 28-day test period. Solutions were changed daily and put in vials with cover that prevent evaporation of staining solutions. Spectrophotometric analysis was made 7, 14, 21 and 28 days after the beginning of the experimentation. Before each measurement, the specimens were gently rinsed with distilled water and air-dried.

### 4.3. Color Testing

A colorimetric evaluation according to the CIE L*a*b* system was performed by a blind trained operator at 5 experimental periods: immediately after light-polymerization and at 7, 14, 21, 28 days of the staining process. The control samples have not been subjected to the staining process. Color of the specimens was measured with a spectrophotometer (SP820λ; Techkon Gmbh, Konig-Stein, Germany) against a black background in order to simulate the absence of light in the mouth against a white background. All specimens were chromatically measured 4 times and the average values were calculated; then each color parameter for each specimens of the same shade was averaged. The CIE 1976 L*a*b* color system is used for the determination of color differences. The L* value refers to “lightness”; the higher is the L value, and it is the lightness (a value of 100 corresponds to perfect white and that of zero to black). CIE L* a* b* values are called the “chromaticity coordinates”; “a*” shows red color on positive values and green color on negative values; “b*” shows yellow color on positive values and blue color on negative values. The total color differences (ΔEab*) were calculated as follows:

ΔEab* = [(ΔL*)2 + (Δa*)2 + (Δb*)2]1/2

where L* is lightness, a* is green–red component (−a* = green; +a* = red) and b* is blue–yellow component (−b* = blue; +b* = yellow). The color measurements of the experimental groups were compared with those of the control group. 

### 4.4. Statistical Analysis

Differences in color change by the immersion protocols were calculated and a statistical analysis was performed using statistical software (Stata 12; College Station, TX, USA). Descriptive statistics that included mean, standard deviation, median, and minimum and maximum values were calculated for each CIE L*a*b* parameter. The Shapiro–Wilk test was applied to assess the normality of the distribution of each CIE L*a*b* parameter. A 2-way parametric analysis of variance test (ANOVA) was applied to determine whether significant differences existed among the groups. The Tukey test was used as post-hoc. A preset alpha level of .05 was used for all statistical analyses. Adjunctive analysis with a paired *t*-test was applied to each CIE L*a*b* parameter when restorative materials were immersed in coffee.

## 5. Conclusions

Long-term exposure to some food dyes (coffee in particular) can significantly affect the color stability of modern esthetic restorative materials regardless of materials’ different compositions.

## Figures and Tables

**Table 1:** Mean ± standard deviation of ΔE calculated from mean ΔL*, Δa* Δb* values for each composite material at the end of the study. Groups with the same superscript letter (“^a^” to “^o^”) are not significantly different (*p* > 0.05).

Composite	Control	Wine	Coffee
Gradia Direct	16.3 ± 1.2 ^a^	41.1 ± 0.7 ^e^	28 ± 1.9 ^i^
Filtek Supreme XTE	21.9 ± 3.1 ^b,o^	39.8 ± 8.5 ^e^	23.4 ± 5.4 ^l,o^
Ceram-X Universal	12.4 ± 0.12 ^c^	28.2 ± 1.8 ^f^	15.2 ± 1.5 ^m^
G-aenial	13.1 ± 0.7 ^c,p^	30.3 ± 3.4 ^f^	16.8 ± 2.9 ^m,p^
Essentia	7.1 ± 1.1 ^d^	33.2 ± 9.1 ^f^	14.2 ± 3 ^m^
Admira Fusion	7.8 ± 0.3 ^d^	51.3 ± 2.8 ^g^	11.9 ± 1.1 ^n^
Estelite	6.1 ± 0.7 ^d^	22.9 ± 0.5 ^h^	11.8± 0.8 ^n^

**Table 2:** Esthetic restorative materials used in this study.

Material	Type	Composition	Filler Content % (*w*/*w*)	Manufacturer	Lot Number
1. Gradia Direct Anterior	Microfilled composite	Matrix: urethanedimethacrylate (UDMA), dymethacrylate camphoroquinone Filler: fluoro-alumino-silicate glass silica powder	73 (*w*/*w*)	GC Corporation, Tokyo, Japan	150527A
2. Filtek Supreme XTE	Nanofilled composite	Matrix: Bis-phenol A diglycidylmethacrylate (Bis-GMA), triehtylene glycol dimethacrylate (TEGDMA), urethane dimethacrylate (UDMA), bis-phenol A polyethylene glycol diether dimethacylate Filler: silica nanofillers (5–75 nm), zirconia/silica nanoclusters (0.6–1.4 µm)	78.5 (*w*/*w*)	3M ESPE, St Paul, MN, USA	N748173
3. Ceram.X Universal	Nanoceramic composite	Matrix: methacrylate modified ploysiloxane, dimethacylate resin, fluorescent pigment, UV stabilizer, stabilizer, camphoroquinone, ethyl–4 (dymethylamino) benzoate, iron oxide pigments, aluminium sulfo silicate pigments. Filler: Barium-aluminium borosilicate glass (1.1–1.5 µm), Methacrylate functionalized silicon dioxide nano filler (10 nm)	76 (*w*/*w*)	Dentsply De Trey, Konstanz, Germany	1507000661
4. G-aenial	Microfilled hybrid composite	Matrix: urethane dimethacrylate (UDMA), dimethacrylate co-monomers. Filler: silica, strontium, lanthanoid fluoride (16–17 µm), silica (>100 nm) fumed silica (<100 nm)	76 (*w/w)*	GC Corporation, Tokyo, Japan	151029A
5. Essentia enamel	Microfilled hybrid composite	Matrix : urethane dimethacrylate (UDMA), Bis-MEPP, Bis-EMA, Bis-GMA, TEGDMA Filler: prepolymerised fillers, barium glass, fumed silica	81 (*w/w)*	GC Corporation, Tokyo, Japan	151109C
6. Admira Fusion	Nanohybrid Ormocer based composite	Matrix: resine Ormocer Filler: silicon oxide nano filler, glass ceramics filler (1 µm)	84 (*w*/*w*)	Voco, Cuxhaven, Germany	1601121
7. Estelite	Supra-nano spherical hybrid composite	Matrix: Bis-phenol A diglycidylmethacrylate (Bis-GMA), Bisphenol A polyethoxy methacrylate (Bis-MPEPP), triehtylene glycol dimethacrylate (TEGDMA), urethane dimethacrylate (UDMA) Filler: Supra-nano Spherical filler (200 nm spherical SiO2-ZrO2), Composite Filler (include 200 nm spherical SiO2-ZrO2).	82 (*w*/*w*)	Tokuyama Dental corporation, Taitou-kuTokyo, Japan	6.6 × 10^17^
